# Chronic Childhood Peer Rejection is Associated with Heightened Neural Responses to Social Exclusion During Adolescence

**DOI:** 10.1007/s10802-015-9983-0

**Published:** 2015-03-12

**Authors:** Geert-Jan Will, Pol A. C. van Lier, Eveline A. Crone, Berna Güroğlu

**Affiliations:** 1 Institute of Psychology, Leiden University, Wassenaarseweg 52, 2333 AK Leiden, The Netherlands; 2 Leiden Institute for Brain and Cognition (LIBC), Leiden University Medical Center, P.O. Box 9600, 2300 RC Leiden, The Netherlands; 3 Department of Developmental Psychology, VU University, Van der Boechorststraat 1, 1081 BT Amsterdam, The Netherlands; 4 Department of Psychology, University of Amsterdam, Weesperplein 4, 1018 XA Amsterdam, The Netherlands

**Keywords:** Anterior cingulate cortex, Cyberball, fMRI, Ostracism, Peer relations, Peer status

## Abstract

This functional Magnetic Resonance Imaging (fMRI) study examined subjective and neural responses to social exclusion in adolescents (age 12–15) who either had a stable accepted (*n* = 27; 14 males) or a chronic rejected (*n* = 19; 12 males) status among peers from age 6 to 12. Both groups of adolescents reported similar increases in distress after being excluded in a virtual ball-tossing game (Cyberball), but adolescents with a history of chronic peer rejection showed higher activity in brain regions previously linked to the detection of, and the distress caused by, social exclusion. Specifically, compared with stably accepted adolescents, chronically rejected adolescents displayed: 1) higher activity in the dorsal anterior cingulate cortex (dACC) during social exclusion and 2) higher activity in the dACC and anterior prefrontal cortex when they were incidentally excluded in a social interaction in which they were overall included. These findings demonstrate that chronic childhood peer rejection is associated with heightened neural responses to social exclusion during adolescence, which has implications for understanding the processes through which peer rejection may lead to adverse effects on mental health over time.

**Electronic supplementary material:**

The online version of this article (doi:10.1007/s10802-015-9983-0) contains supplementary material, which is available to authorized users.

## Introduction

Children and adolescents who are rejected by peers suffer from widespread impairments in mental health that can persist across development (Ladd 2006; Ladd and Troop-Gordon 2003; Prinstein and Aikins 2004). A potential mechanism through which a rejected status among peers leads to mental health problems is a heightened emotional and neural reactivity to negative treatment that accompanies a rejected status (e.g., being ignored, harassed, excluded). For example, boys with a rejected status who are more distressed by a rejection experience have more externalizing behavioral problems than boys with a rejected status who show lower levels of reactive distress (Sandstrom et al. 2003). Similarly, adolescents who display enhanced neural responses to social exclusion are more likely to exhibit depressive symptoms 1 year later (Masten et al. 2011). Given that children’s social experiences in peer groups play a crucial role in shaping their perceptions and expectations about future social interactions (Crick and Dodge 1994; Ladd et al. 2014; London et al. 2007), sustained exposure to either high or low levels of peer group rejection is likely to have an impact on how adolescents respond to negative peer treatment, such as social exclusion. Therefore, we examined subjective and neural responses to social exclusion in adolescents who either had a stable accepted or a chronic rejected status among their classmates across six elementary school grades.

### Chronic Peer Group Rejection and Psychosocial Development

Adverse adjustment outcomes resulting from peer group rejection have been well documented. Peer group rejection has proven to be reliably assessed by asking children to nominate the classmates they like and dislike (Asher and Coie 1990; Bukowski et al. 2000; Jiang and Cillessen 2005; Parker and Asher 1987). Children who receive many negative (dislike) nominations and who receive few positive (like) nominations are classified as having a rejected status (Asher and Dodge 1986; Bukowski et al. 2000). A rejected status has been found to be highly stable across time and across different social contexts (Coie and Kupersmidt 1983; Hardy et al. 2002; Jiang and Cillessen 2005). Moreover, a chronic rejected status has been prospectively linked to an array of impairments in daily life, ranging from poor academic achievement (DeRosier et al. 1994) to an increased incidence of both internalizing (Ladd and Troop-Gordon 2003) and externalizing behavior problems (Sturaro et al. 2011).

Transactional models of peer rejection posit that such impairments in daily life arise from a sustained pattern of reciprocal interactions between peers expressing their dislike and the way a rejected child responds to being disliked (Coie 1990). For example, repeated exposure to rejection experiences (e.g., social exclusion) may elicit negative emotions (e.g., anger at exclusion) resulting in aggressive reactions, which in turn could trigger repeated instances of exclusion by the peer group ultimately giving rise to externalizing problems (Coie 2004; Dodge et al. 2003). Internalizing problems have been hypothesized to arise from a similar developmental cascade in which repeated exposure to rejection experiences may amplify negative emotions (e.g., sadness, distress), which in turn heighten anxiety, lead to withdrawal or bolster psychological processes that underlie the development of internalizing disorders (e.g., low self-esteem, lower levels of trust in others) (Ladd et al. 2014; Troop-Gordon and Ladd 2005). As such, a rejected status could be maintained across development through a heightened emotional or neural reactivity to negative treatment such as social exclusion. Understanding the mechanisms underlying the maintenance of a rejected status can aid in understanding why some children are able to deal with episodes of peer rejection without much difficulty whereas others become trapped in a pattern of sustained rejection and associated impairments in daily life (Sandstrom 2004; Sandstrom and Coie 1999).

### Social Exclusion: Distress and Neural Correlates

Social exclusion is highly distressing and immediately threatens fundamental human needs, such as our need to belong, our need for control over our (social) environment and our needs for self-esteem and a meaningful existence (Baumeister and Leary 1995; Williams 2007). Relationships with peers are vital to satisfying these needs across the lifespan (Ladd 1999; Rubin et al. 2006) and therefore we hypothesized that childhood peer acceptance and rejection have an impact on the extent to which these needs are threatened by social exclusion in adolescence. An experimental design which has proven to be a reliable paradigm to elicit exclusion-related distress is a virtual ball-tossing game called Cyberball (Williams et al. 2000). After being ostensibly excluded by two peers in Cyberball, children, adolescents and adults consistently report heightened levels of distress in the form of higher levels of negative mood (e.g., sadness and anger) and a decreased satisfaction of the need to belong, the need for control, self-esteem, and the need for a meaningful existence (Abrams et al. 2011; Gunther Moor et al. 2012; van Beest and Williams 2006).

Functional Magnetic Resonance Imaging (fMRI) studies using the Cyberball game have identified a network of brain regions involved in processing exclusion-related distress of which three regions are most consistently found: the anterior cingulate cortex (ACC), the anterior insula and the ventrolateral prefrontal cortex (vlPFC) (Cacioppo et al. 2013; Eisenberger 2012; Rotge et al. 2014). Higher levels of need threat have been associated with higher levels of activation in the anterior insula and dorsal, ventral and subgenual regions of the ACC (Bolling et al. 2011; Eisenberger et al. 2003; Gunther Moor et al. 2012; Masten et al. 2009). Consistent with the ACC and insula’s involvement in processing conflict and (negative) emotions, these findings suggest that the ACC and insula are involved in processing the distress caused by exclusion. Negative associations have been found between self-reported need threat and activation in the vlPFC, suggesting that the vlPFC is involved in regulating the distress caused by exclusion (Bolling et al. 2011; Eisenberger et al. 2003; Masten et al. 2009).

Notably, fMRI studies have also highlighted that activity in these brain regions during exclusion may be enhanced or attenuated depending on individual or social factors. Chronic peer group rejection may be one such social factor, and individual factors identified in previous research (e.g., sensitivity to rejection, an anxious attachment style, perceived social support) are likely characteristic of adolescents who have experienced chronic rejection. That is, higher levels of ACC activity during social exclusion have been observed in adolescents who reported to be more sensitive to rejection (Masten et al. 2009) and in adults with an anxious attachment style characterized by a vigilance to cues of rejection (DeWall et al. 2012). Furthermore, adults who perceived their daily social interactions to be more comforting and supportive showed dampened ACC activation during exclusion (Eisenberger et al. 2007) and young adults who spent more time with friends during late adolescence showed a similar pattern of reduced ACC and insula activity during exclusion (Masten et al. 2012). Taken together, these findings suggest that people who are more sensitive to rejection or who have lower levels of (perceived) social support display higher levels of activity in brain regions involved in processing the distress caused by social exclusion. Consequently, it is likely that adolescents with a history of peer rejection, who are often more sensitive to rejection (London et al. 2007) and perceive lower levels of peer social support (Ladd et al. 2014) than adolescents with a history of peer acceptance, display enhanced neural responses in the ACC or anterior insula when they are excluded.

### The Current Study

To test the hypothesis that adolescents with a history of chronic peer rejection display enhanced neural responses to social exclusion compared to stably accepted adolescents, we recruited participants from a sample of adolescents who were followed yearly in their classrooms since they were 6 years old. We invited participants who were, across six elementary school grades, consistently nominated by their peers to be liked and almost never disliked (i.e., those with a stable high social preference among their peers, or, stably accepted adolescents) and participants who were consistently disliked and almost never liked (i.e., those with a chronic low social preference, or, chronically rejected adolescents), and examined differences in their subjective and neural responses to exclusion in Cyberball using whole-brain fMRI analyses. Based on previously found negative associations between concurrent social preference and self-reported distress after a mild social rejection experience (Sandstrom et al. 2003), we hypothesized that adolescents with a history of chronic rejection would report higher levels of distress (i.e., lower mood and need satisfaction) after exclusion compared to adolescents with a history of stable acceptance. We further hypothesized that adolescents with a history of peer rejection would show heightened activity in brain regions previously linked to the distressing aspect of social exclusion (e.g., ACC and anterior insula) compared to adolescents with a history of stable acceptance. To test whether chronically rejected adolescents would also show neural reactivity indicative of a hypervigilance to cues of potential rejection, we investigated neural responses to events during which participants did not receive the ball in a social interaction in which they were overall included (i.e., incidental exclusion).

## Method

### Participants

The current study formed the eighth wave of a longitudinal study on the impact of elementary school social experiences on child behavioral, emotional and academic outcomes where participants were followed between the ages of 6 and 12. A total of 1189 participants were followed annually from first to sixth grade of elementary school. Each year participants filled out a peer-nomination procedure (unlimited nominations), in which participants were asked to name the peers in their class who they liked most and liked least. An average social preference score (liked most minus liked least nominations) across the six waves was computed. Subsequently, participants were identified as chronically rejected if they were in the lower 10th percentile or as stably accepted if they were in the upper 10th percentile of that 6-year average social preference. By using a 10% threshold it was ensured that none of the chronically rejected adolescents were ever classified as sociometrically popular and none of the stably accepted adolescents were ever classified as having a rejected status across the six waves. Correlation coefficients between social preference scores of adjacent years ranged from 0.67 to 0.70 (all *p*’s < .001), which is comparable to those reported in other studies (Salmivalli and Isaacs 2005; Vitaro et al. 2007). Participation rates of in the classrooms across cohorts and six annual assessments ranged from 88 to 99 %, indicating that participation rates in the classroom nomination assessments were above recommended thresholds (Marks et al. 2013).

Based on these criteria, 219 adolescents were eligible for participation in the fMRI study. Of these youths, recent full contact information was available for 131 adolescents, who were subsequently approached for participation in the fMRI study. Twenty adolescents were excluded because they were left-handed (*n* = 4), had an autism spectrum disorder (*n* = 1) or had braces (*n* = 15). Seven adolescents could not be reached. Of the remaining 104 candidate participants, 47 adolescents and their parents agreed to participate in the fMRI study. Those who chose not to participate in the fMRI study (*n* = 57) did not differ from those who were scanned with respect to average social preference, age, gender, and average levels of anxiety and conduct problems across 6 years of elementary school (all *p*’s > .19). A radiologist reviewed all anatomical scans, and one participant was excluded from the analyses due to an anomaly. Two participants were excluded from the analyses because their head movement parameters exceeded 1 voxel (3 mm) in at least one direction.

The remaining 44 participants had a mean age of 14.0 years (*SD* = 0.70; 26 males). Twenty-seven adolescents met our criteria for a history of stable peer acceptance (*M* age = 14.0; *SD* = 0.77; 14 male) and 17 for a history of chronic peer rejection (*M* age = 14.0; *SD* = 0.56; 12 male). All participants indicated to be healthy and reported no contraindications for MRI (e.g., no head injuries, no history of neurological or psychiatric disorders), except for four participants with a history of chronic peer rejection who were diagnosed with Attention-Deficit Hyperactivity Disorder (ADHD). Three of these participants with ADHD were on a stable dose of methylphenidates, but were medication-free on the day of scanning and the preceding day. One participant was on medication during scanning. The two groups of adolescents did not differ in gender, age, pubertal status, ethnicity, or IQ (all *p*’s > .15; see Table 1). Chronically rejected adolescents had higher average levels of anxiety and conduct problems across 6 years of elementary school and they had lower levels of social competence at the moment of scanning than stably accepted adolescents (all *p*’s < .05). Researchers and research assistants were familiar with the recruitment procedure based on childhood histories of acceptance and rejection, but were not informed about individual participants’ peer status history to ensure blind assessments during data collection. All participants and their parents gave informed consent for the study. The medical ethical committee of the VU University Medical Center approved the longitudinal study and the MRI study was approved by the Leiden university medical ethical committee. After scanning, participants filled out a battery of questionnaires and were debriefed. Participants received a monetary compensation for participation and small gifts.

**Table 1:** Participant characteristics

Characteristics and questionnaires	Group, Mean (SD)		*p*-value^a^
	Chronically rejected (*n* = 17)	Stably accepted (*n* = 27)	
Mean Social Preference^*b*^ (selection variable)	−1.62 (0.52)	1.16 (0.18)	<0.001
Gender (% Male)	70.6	51.9	0.22
Age	13.98 (0.77)	14.04 (0.58)	0.78
Pubertal status (PDS)			
• Males	2.44 (0.77)	2.19 (0.59)	0.36
• Females	3.17 (0.26)	2.72 (0.63)	0.15
Race/Ethnicity (% Caucasian)	100%	96.3%	0.44
IQ (WISC Similarities and Block Design)	95 (12.68)	100 (10.24)	0.16
Current social competence (parent reported)	4.59 (0.62)	5.40 (0.57)	<0.001
Anxiety during elementary school (teacher reported)^b^	0.41 (0.80)	−0.31 (1.01)	<0.05
Conduct problems during elementary school (teacher reported)^b^	0.81 (1.39)	−0.67 (0.52)	<0.001

^a^All *p*-values obtained using t tests except for race and gender (Chi-square tests)

^b^Average across 6 years of elementary school, Z-standardized

### fMRI Task: Cyberball

Participants were given a cover story, in which they were told that they were about to perform a mental visualization task and that this would be investigated by means of an online ball-tossing game (Williams et al. 2000). Accordingly, they were asked to imagine what the other players looked like, what kind of personalities they would have and in what kind of weather conditions the game would be played (Williams 2007). It was explained that the players were unfamiliar peers and that they would be connected through the Internet. Unbeknownst to the participants, the behavior of the other players in Cyberball was preprogrammed. The other players in the game were depicted as cartoon characters with their names depicted below them (1 male; 1 female). The participants were represented by a hand in the middle of the screen and they could throw the ball to the left or right player with a button press of the index finger of the corresponding hand.

Participants first played an inclusion condition in which each player received the ball an equal amount of times (10 out of 30 throws). After filling out short questionnaires assessing mood and need satisfaction in the scanner (see below), participants played the exclusion condition where, after receiving the ball once at the start of the game and throwing it to one of the other players, they did not receive a single ball for the remainder of the game (28 out of 30 throws). Scans were acquired during two separate runs each lasting about 3 min. Participants’ throws were self-paced, ball throws of the other players were preceded by a random jitter interval (100–4000 ms) and it took 2 s before each throw reached the designated player. During debriefing, we administered a funneling suspicion probe about the authenticity of the players in Cyberball consisting of three open-ended questions (see online Electronic supplementary material). The number of participants who raised suspicions did not differ between the two groups (7 stably accepted vs. 6 chronically rejected adolescents), *χ* ^2^(1) = 0.4, *p* = .51.

### Questionnaires: Mood and Need Satisfaction

To assess exclusion-related distress we used self-report measures of mood and need satisfaction (Gunther Moor et al. 2012; Lelieveld et al. 2013; Sebastian et al. 2010; Will et al. 2014). Mood and need satisfaction were assessed at three time points: 1) immediately after inclusion, 2) immediately after exclusion and 3) approximately 30 min after exclusion (when participants came out of the scanner).

The Need Satisfaction questionnaire consisted of eight items taken from the Need Threat Scale (van Beest and Williams 2006), with two questions assessing each of the following four needs: belonging, self-esteem, control and meaningful existence (see Supplementary Table 1 online). All need satisfaction items were rated on a scale from 1 (*not at all*) to 5 (*very much*) and negative items were recoded. Higher scores on this measure thus reflect satisfaction of these needs and lower scores reflect the threat of these needs. The mood questionnaire consisted of eight mood items (feeling good, bad, happy, sad, relaxed, tense, friendly and unfriendly (see Supplementary Table 1 online). All mood items were rated on a scale from 1 (*not at all*) to 5 (*very much*) and negative mood items (bad, sad, tense, unfriendly) were recoded.

Internal consistency of the need satisfaction scale proved to be good (Cronbach’s α = 0.78) and therefore, consistent with previous studies using Cyberball (van Beest and Williams 2006; Williams et al. 2000), the four need scales were averaged to create an overall index of need satisfaction at each time-point, i.e., after inclusion, after exclusion and post-scanning. Internal consistency of the mood scale was acceptable (Cronbach’s *α* = 0.67) and the four mood constructs were averaged to create an overall index of mood at each time-point.

### fMRI Data Acquisition

Participants were first familiarized with the scanner environment through the use of a mock scanner. Scans were acquired using a 3T Philips Achieva MRI system at the Leiden University Medical Center. Stimuli were projected onto a screen located at the head of the scanner bore using Authorware. Participants viewed the screen via a mirror mounted on the head coil. Foam inserts that surrounded the head were used to minimize head movement. First, we obtained a localizer scan for each participant. Second, T2*-weighted Echo-Planar Images (EPI) were obtained (repetition time (TR) = 2.2 s, echo time (TE) = 30 ms, sequential acquisition, 38 slices of 2.75 mm, field of view (FOV) = 220 × 220 × 114.68 mm) during two functional runs: Cyberball inclusion and exclusion. The first two volumes of each functional run were discarded to allow for equilibration of T1 saturation effects. Finally, we obtained a high-resolution 3D T1-FFE scan for anatomical reference (TR = 9.76 ms, TE = 4.59 ms, flip angle = 8°, 140 slices, voxel size = 0.875 × 0.875 × 1.2 mm voxels, FOV = 224 × 177 × 168 mm) after the functional runs.

### fMRI Data Analysis

MRI data were preprocessed and analyzed using SPM8 statistical parametric mapping image analysis software (Wellcome Trust Centre for Neuroimaging, University College London). Functional images were slice-time corrected, realigned, co-registered to individual structural T1 scans, normalized to a T1 template, and spatially smoothed using an 8 mm, full-width at half-maximum isotropic Gaussian kernel. The normalization algorithm, resampled the volumes to 3 mm cubic voxels using a 12-parameter affine transformation and a nonlinear transformation involving cosine basic functions. All results are reported in MNI305 stereotactic space.

We analyzed the fMRI data using an event-related design based on previous studies (Gunther Moor et al. 2012; Will et al. 2014). Data were modeled as zero-duration events at the onset of a ball-toss and convolved with a canonical hemodynamic response function (HRF). Statistical analysis was carried out using a general linear model. Regressors were defined for three Cyberball events (throwing, receiving or a ball-toss between the two other players) and were analyzed separately for the inclusion game and the exclusion game. The model contained a basic set of cosine functions that high-pass-filtered the data and a covariate to control for run effects. The least-squares parameter estimates of the height of the best-fitting canonical HRF for each condition separately were used in pair-wise contrasts at the subject level. The resulting contrast images were submitted to group analyses where participants were treated as a random effect. Subsequently, we performed whole-brain one-tailed *t-*tests to examine the neural correlates of social exclusion and incidental exclusion across the sample. For group comparisons, contrast images were entered into separate second-level analyses for each contrast of interest, where peer status history (chronically rejected vs. stably accepted) was the between-subjects variable in whole-brain independent samples *t*-tests. Results were considered significant at an uncorrected threshold of *p* < .001 with a minimum cluster size of 10 contiguous voxels to balance between Type 1 and Type 2 errors (Lieberman and Cunningham 2009). We also report which results remain significant using a whole-brain voxel-wise false discovery rate (FDR) correction (*p* < .05, > 10 voxels). We used the Marsbar toolbox (Brett et al. 2002); http://marsbar.sourceforce.net/) to extract activity in functional regions of interest.

## Results

### Self-Reported Distress

**Fig. 1:**
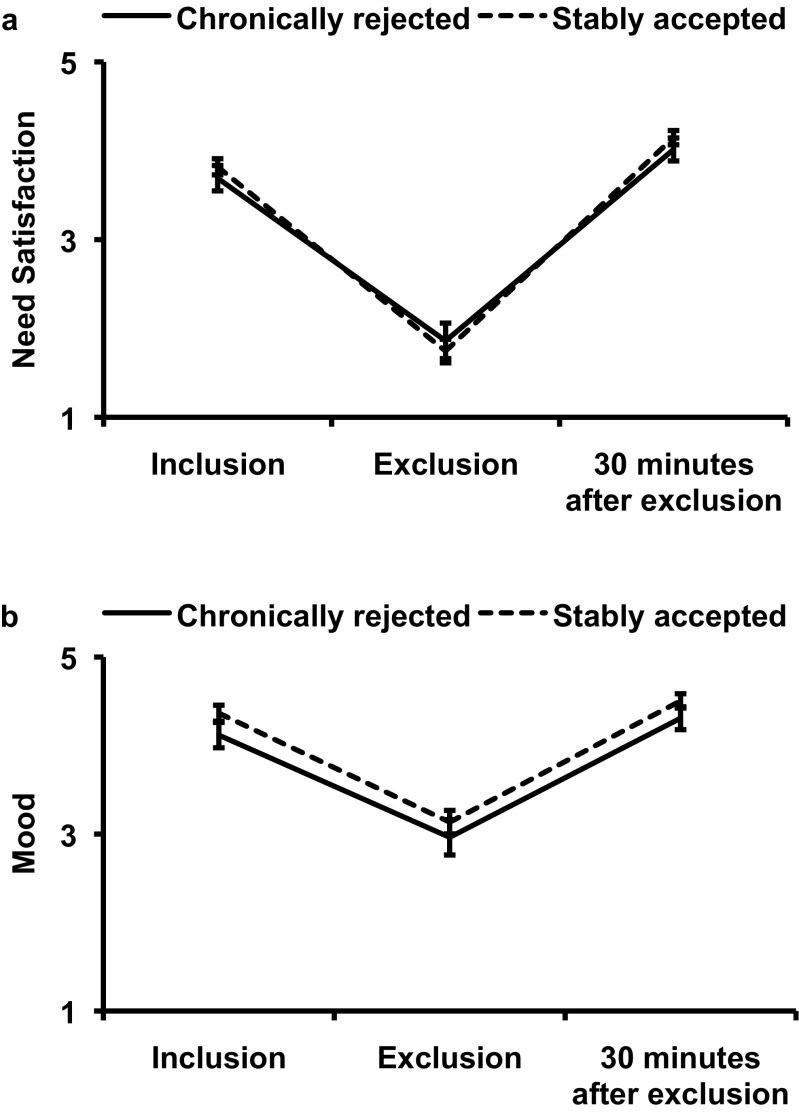
**a** Mean levels of a composite score of self-reported satisfaction of fundamental human needs (belonging, self-esteem, control and meaningful existence) assessed immediately after inclusion, exclusion and 30 min after exclusion. **b** Mean levels of a composite score of mood assessed immediately after inclusion, exclusion and 30 min after exclusion (*error bars* represent standard errors of the mean)

A repeated measures ANOVA with time point (3 levels: inclusion, exclusion, and 30 min after exclusion) as within-subjects factor for the composite score of need satisfaction and peer status history (2 levels: chronically rejected vs. stably accepted) as a between-subjects factor yielded a main effect of time point, *F*(2, 84) = 221.73, *p* < .001, η_*p*_^*2*^ = 0.84. Follow-up pairwise comparisons showed that need satisfaction assessed immediately following exclusion was lower than need satisfaction after inclusion (*p* < .001) and 30 min after exclusion (*p* < .001) (see Fig. 1a). The interaction effect between time point and peer status history was not significant (*p* = .49), indicating that effects of social exclusion on need satisfaction were similar for stably accepted and chronically rejected adolescents.

Similarly, a repeated measures ANOVA with time point (3 levels: after inclusion, after exclusion and 30 min after exclusion) as within-subjects factor for overall mood with peer status history (2 levels: chronically rejected vs. stably accepted) as a between-subjects factor yielded a main effect of time point, *F*(2, 84) = 98.24, *p* < .001, η_*p*_^*2*^ = 0.71. Follow-up pairwise comparisons showed that mood assessed immediately following exclusion was lower than after inclusion (*p* < .001) and 30 min after exclusion (*p* < .001) (see Fig. 1b). The interaction effect between time point and peer status history was not significant (*p* = .87), indicating that effects of social exclusion on mood were similar for stably accepted and chronically rejected adolescents.

### Neuroimaging Results

#### Neural Responses to Social Exclusion Across the Sample

Before we tested for differences in brain responses between chronically rejected and stably accepted adolescents, we first investigated the neural correlates of social exclusion across the whole sample. Three contrasts were used: two examining the neural correlates of social exclusion and a third contrast examining the neural correlates of incidental exclusion.

**Fig. 2:**
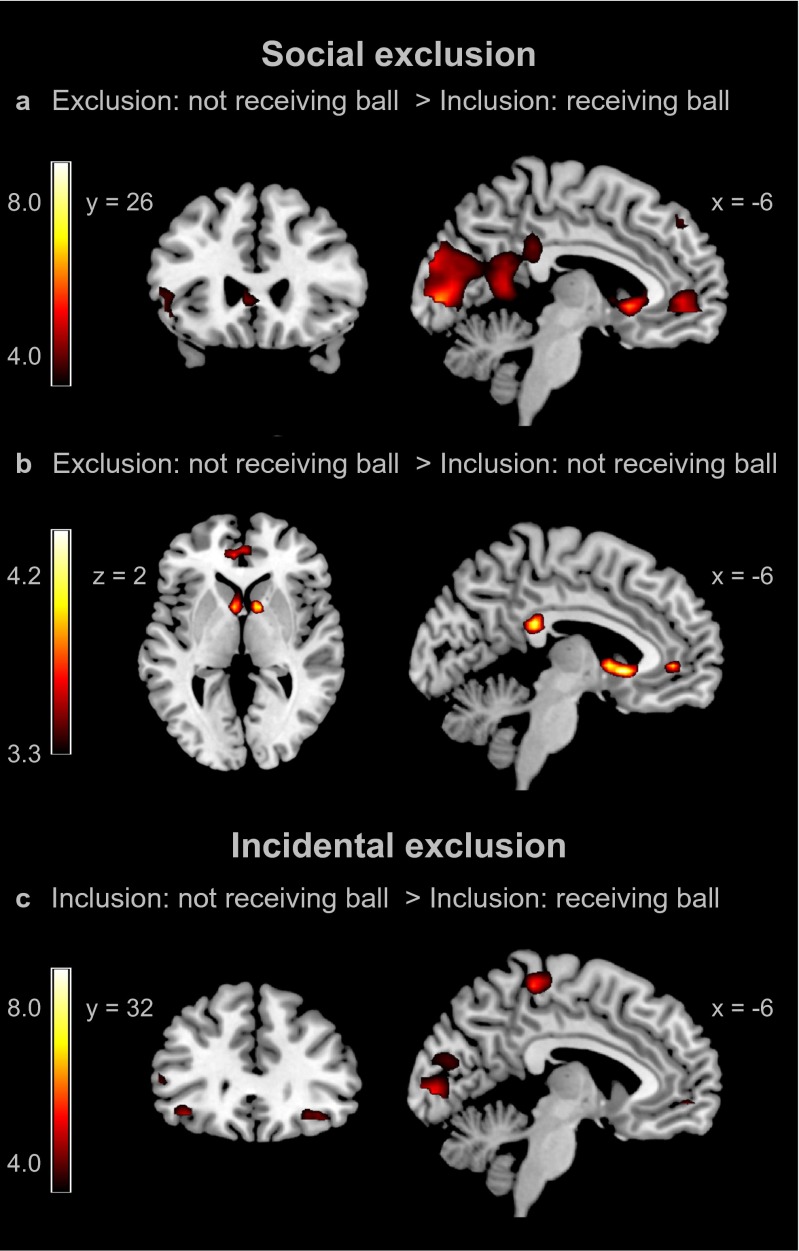
Whole-brain results for the Cyberball interaction collapsed across peer status groups. **a** Social exclusion 1: Not receiving the ball during the exclusion game > receiving the ball during inclusion game. **b** Social exclusion 2: Not receiving the ball during the exclusion game > Not receiving the ball during inclusion game. **c** Incidental exclusion: Not receiving the ball during the inclusion game > receiving the ball during inclusion game

The first contrast, which compared activation on trials where participants did not receive the ball in the exclusion game with trials where participants received the ball in the inclusion game (Exclusion: not receiving the ball > Inclusion: receiving the ball), resulted in activation in a set of brain regions (Fig. 2a), including ventral ACC/medial PFC (vACC/mPFC; peak voxel of cluster at -12, 47, 1), striatum (peak at -6, 17, -2), bilateral vlPFC (peaks at 27, 32, -11 and -45, 32, -8) and the dorsomedial PFC (peak at -6, 47, 46).

The second contrast, which compared activation on trials where participants did not receive the ball in the exclusion game with trials where they did not receive the ball in the inclusion game (Exclusion: not receiving the ball > Inclusion: not receiving the ball), resulted in activation in the striatum (peak at -6, 17, -2) and the vACC (peak at -6, 44, 1) (Fig. 2b).

To identify the neural regions associated with incidental exclusion, we compared activation on trials where participants did not receive the ball during the inclusion game with trials where participants received the ball during the same game (Inclusion: not receiving the ball > Inclusion: receiving the ball). This whole brain contrast resulted in increased activation in several brain regions, including bilateral inferior frontal gyrus (IFG; peaks at 36, 32, -11 and -39, 32, -11), medial PFC (peak at -9, 50, -5) and left vlfPFC (peak at -54, 29, 7) (see Fig. 2c). All significant clusters (uncorrected and FDR corrected) are reported in Supplementary Table 2 online.

#### Neural Responses to Social Exclusion Associated with Peer Status History

**Fig. 3:**
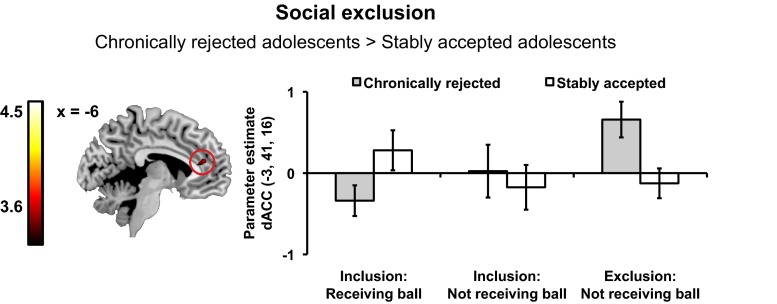
Chronically rejected adolescents showed increased activation in the dorsal anterior cingulate cortex (dACC; -3, 41, 16) during social exclusion compared to stably accepted adolescents. Subject-level contrast values in this region of the dACC were extracted for events on which participants received the ball during the inclusion game, when they did not receive the ball during the inclusion game and when they did not receive the ball during the exclusion game and plotted to facilitate interpretation

**Fig. 4:**
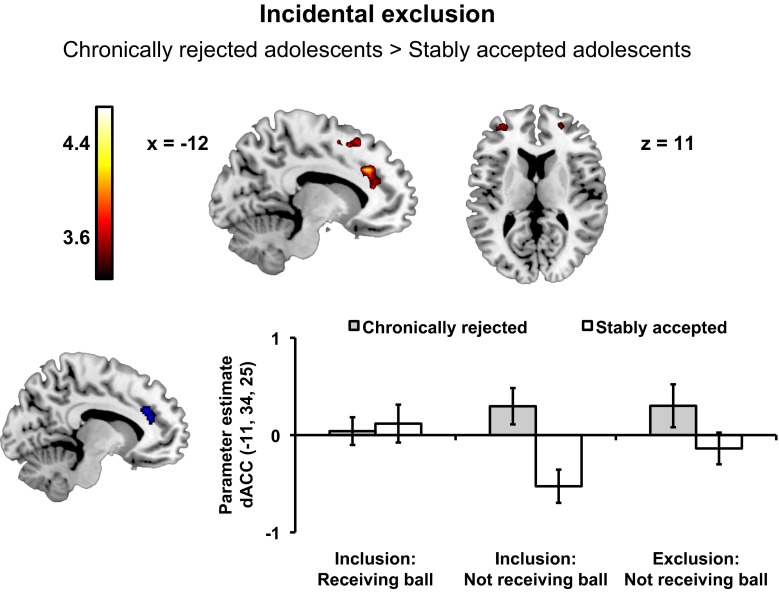
Chronically rejected adolescents, compared to stably accepted adolescents, showed increased activation in the pre-supplementary motor area (-9, 23, 46), dorsal anterior cingulate cortex (dACC; -15, 29, 31) extending into left anterior prefrontal cortex (aPFC; -36, 50, 13), and right aPFC (24, 50, 13) during incidental exclusion. Subject-level contrast values in this region of the dACC (cluster of activation masked with an anatomical ROI of the ACC from the Automated anatomical labeling ROI library (Tzourio-Mazoyer et al. 2002) were extracted for events on which participants received the ball during the inclusion game, when they did not receive the ball during the inclusion game and when they did not receive the ball during the exclusion game and plotted to facilitate interpretation

To examine differences in brain responses between adolescents with a history of stable acceptance or chronic rejection, we ran whole-brain independent samples *t*-tests on all three contrasts outlined above. Chronically rejected adolescents showed increased activation in dACC (peak at -3, 41, 16) when they were excluded. That is, compared to stably accepted adolescents, chronically rejected adolescents showed higher dACC activity on events where they did not receive the ball during the exclusion game compared with events where they received the ball in the inclusion game (Chronically rejected adolescents [Exclusion: not receiving the ball - Inclusion: receiving the ball] > Stably accepted adolescents [Exclusion: not receiving the ball - Inclusion: receiving the ball]) (see Fig. 3). Additionally, differences in neural responses to incidental exclusion were found. Specifically, a whole brain contrast showed that compared to stably accepted adolescents, chronically rejected adolescents showed increased activation in the pre-supplementary motor area (peak at -9, 23, 46), dACC (peak at -15, 29, 31) extending into left anterior prefrontal cortex [aPFC; peak at -36, 50, 13]), and right aPFC (peak at 24, 50, 13) on incidental exclusion trials (Chronically rejected adolescents [Inclusion: not receiving the ball - Inclusion: receiving the ball] > Stably accepted adolescents [Inclusion: not receiving the ball - Inclusion: receiving the ball]) (see Fig. 4). A direct comparison between stably accepted and chronically rejected adolescents on the second social exclusion contrast (Exclusion no ball > Inclusion no ball) did not result in activation in regions associated with cognitive or affective processes. No regions showed higher activity in stably accepted adolescents than chronically rejected adolescents in any of the three contrasts. All significant clusters are reported in Table 2.

**Table 2:** Brain regions revealed by whole-brain analyses testing for peer status history differences in the Cyberball game (all thresholded *p* < .001 uncorrected, > 10 voxels)

Anatomical region	L/R	Voxels	z	MNI coordinates		
				x	y	z
Social exclusion: Chronically rejected adolescents > Stably accepted adolescents [Exclusion: not receiving the ball - Inclusion: receiving the ball]						
Postcentral gyrus	L	19	3.60	−45	−19	31
Anterior Cingulate cortex	L	17	3.48	−3	41	16
Superior Temporal gyrus	L	10	3.38	−60	−34	19
Social exclusion: Chronically rejected adolescents > Stably accepted adolescents [Exclusion: not receiving the ball - Inclusion: not receiving the ball]						
Heschl’s gyrus	R	15	3.52	48	−22	10
Incidental exclusion: Chronically rejected adolescents > Stably accepted adolescents [Inclusion: not receiving the ball - Inclusion: receiving the ball]						
Anterior Cingulate cortex	L	148	4.21	−15	29	31
extending into:			3.70	−15	35	19
Middle Frontal gyrus (aPFC)			3.45	−36	50	13
Superior Frontal gyrus/	L	26	3.56	−15	20	52
Supplementary Motor Area			3.49	−9	11	55
Superior Frontal gyrus (aPFC)	R	11	3.40	24	50	13
Supplementary Motor Area	R	16	3.37	9	23	46

L/R = Left/Right; k = cluster size in 3 × 3 × 3 mm voxels; Z = z-score; MNI coordinates = xyz voxel coordinates in MNI space of the peak voxel

## Discussion

The present study investigated differences in subjective and neural responses to social exclusion in adolescents who either had a stable accepted or a chronically rejected status across six elementary school grades. We first replicated previous findings, showing that a brief episode of social exclusion is distressing for adolescents (Gunther Moor et al. 2012; Sebastian et al. 2010) and that social exclusion is associated with activation in brain regions implicated in emotion processing and emotion regulation, such as the dorsal and ventral ACC, medial prefrontal cortex (mPFC), the striatum and vlPFC (Bolling et al. 2011; Gunther Moor et al. 2012; Masten et al. 2009; Sebastian et al. 2011). Our findings extend the literature by showing that differences in sustained patterns of peer group acceptance and peer group rejection during the elementary school period are associated with differential neural processing of social exclusion in adolescence. That is, chronically rejected adolescents showed, in comparison to stably accepted adolescents: 1) increased activation in the dACC when they were excluded, and 2) increased activation in the dACC and aPFC during incidental exclusion events in a social interaction in which they were included.

### Childhood Peer Status and Self-Reported Distress After Exclusion

Our results show that a brief episode of exclusion in Cyberball results in immediate distress in the form of decreased mood and need satisfaction and that chronically rejected adolescents and stably accepted adolescents report similar levels of distress. Our results partially overlap with the results from a previous study that examined individual differences in subjective distress after receiving a video message from another child telling the participants that he/she did not want to play with them (Sandstrom et al. 2003). Consistent with our findings, Sandstrom et al. (2003) found no differences in acute distress reported by accepted and rejected boys. However, their findings indicated that rejected girls reported higher levels of distress compared to accepted girls. Our sample was not large enough to test for such interactions between sex and peer status history in order to examine whether distress differed between chronically rejected and stably accepted girls. Future studies with larger samples could test whether individual differences in self-reported distress associated with a stable high or low peer status might be different for boys and girls.

Additionally, methodological differences between paradigms used to elicit rejection-related distress may account for differences in results. That is, the relatively mild rejection experience in Sandstrom et al. (2003) study could have allowed more room for individual differences in responses compared to the Cyberball paradigm. That is, meta-analyses have shown that exclusion in the Cyberball paradigm very reliably induces distress (large effect sizes of exclusion in Cyberball on mood and need satisfaction; D’s between 1 and 2; Gerber and Wheeler 2009), but also that the self-reports of such distress seem to be less amenable to moderation by individual differences, such as the participant’s sex (Williams and Sommer 1997), their levels of loneliness (Wesselmann et al. 2012) or social anxiety (Zadro et al. 2006). Thus, the strength of the Cyberball paradigm (i.e., its ability to reliably induce distress) might also be a limitation when investigating individual differences. A milder or more ambiguous rejection experience might allow for more variability in responses, which could be related to individual differences such as peer status.

### Childhood Peer Status and Neural Responses to Exclusion

The neuroimaging results show that neural responses to both social exclusion and incidental exclusion differ between adolescents who were chronically rejected and those who had a stable accepted during childhood. Compared to stably accepted adolescents, chronically rejected adolescents showed heightened dACC activity during social exclusion. Our findings are in line with previous work showing enhanced dACC activation during exclusion in adolescents who are more sensitive to rejection (Masten et al. 2009), adults with low self-esteem (Onoda et al. 2010), adults who perceived their daily social interactions to be less comforting and supportive (Eisenberger et al. 2007) and young adults who spent less time with friends during late adolescence (Masten et al. 2012). Combining these previous findings with our results suggest that chronically rejected adolescents show an enhanced neural response to exclusion that they share with people who are more sensitive to rejection, who have lower levels of self-esteem and who have less satisfying social relations.

What could the higher levels dACC activity during exclusion reflect? The ACC is implicated in a wide variety of cognitive and emotional processes including conflict monitoring (Botvinick et al. 2004), expectancy violation (Somerville et al. 2006), physical pain and other negative emotions (Shackman et al. 2011), reactions to being treated unfairly (Sanfey et al. 2003) and social exclusion (Eisenberger et al. 2003). It has been proposed that the ACC is central to a system involved in monitoring the extent to which autonomic/affective signals elicited by salient events interfere with goals or ongoing behavior and therefore require increased attention (Shenhav et al. 2013). Furthermore, it has been put forward that there is a functional dissociation between dorsal and ventral parts of the ACC (Somerville et al. 2006). That is, the dorsal ACC is connected with prefrontal, parietal and motor cortices, and is important for signaling conflict and integrating top-down and bottom-up processes (Shenhav et al. 2013). The ventral ACC is connected to regions involved in generating and processing affect, such as the amygdala, striatum, and anterior insula, and has been implicated in integrating emotional and motivational valence of stimuli (Somerville et al. 2006).

Chronically rejected and stably accepted adolescents did not show differences in ventral ACC activity, suggesting that exclusion is emotionally salient irrespective of childhood peer status. This notion was mirrored by the similarities in self-reported distress after exclusion. Thus, although it could be hypothesized that chronic exposure to negative peer experiences might desensitize children’s reactions to social exclusion, our findings suggest otherwise. Specifically, the finding that chronically rejected adolescents showed increased activation of the dACC compared to stably accepted adolescents suggests that a persistent low status among peers is associated with a neural signal possibly indicating increased conflict or salience associated with being excluded.

Notably, chronically rejected, compared to stably accepted adolescents, showed enhanced activity in dACC and aPFC in response to incidental exclusion, that is, events during which they did not receive the ball in an interaction in which they were overall included. Higher levels of activity in the dACC and aPFC during exclusion in Cyberball have been shown to be associated with higher levels of rejection sensitivity (Masten et al. 2009), which has been defined as “the disposition to defensively (i.e., anxiously or angrily) expect, readily perceive, and overreact to social rejection” (Downey et al. 1998, p. 1074). Enhanced neural responses to not receiving the ball in the inclusion game in brain regions previously linked to a greater sensitivity to rejection suggest that chronically rejected adolescents might be more sensitive to cues of potential exclusion than stably accepted adolescents. Taken together, these findings show that adolescents with a history of chronic rejection exhibit heightened neural responses to actual and incidental exclusion, which could be indicative of a hypersensitivity or hypervigilance to exclusion.

One possible mechanism accounting for this hypersensitivity could be that chronically rejected adolescents have been exposed to higher levels of negative peer treatment similar to the treatment in Cyberball (being ignored or excluded) than the stably accepted adolescents. Although peer group rejection has been found to be predictive for experiencing peer victimization, including relational victimization (e.g., being left out or excluded from peer activities) (Salmivalli and Isaacs 2005; van Lier and Koot 2010), there are large individual differences in the extent to which children with a rejected status are victimized; both in terms of frequency and severity (Boivin et al. 1995; Boulton 1999). Future studies should examine individual differences related to chronic exclusion/victimization using peer nominations of being excluded/victimized in a larger sample of chronically rejected adolescents. Such endeavors can shed light on the question of whether neural responses to social exclusion are particularly pronounced in adolescents who have been chronically excluded or victimized.

### Limitations

Several limitations to the current study warrant consideration. First, although our study is the first demonstration of differences in neural responses to exclusion between adolescents with a history of stable peer acceptance and those with a history of chronic peer rejection, we cannot conclude that these differences are the result of their respective peer status histories. Although the more pronounced brain responses among chronically rejected adolescents could plausibly be attributed to their manifest social experiences, we cannot rule out that such differences were already present before elementary school and their emerging peer status. Future longitudinal studies investigating whether changes in peer status are linked to changes in brain response may shed more light into the question of direction of effects.

Second, our results are based on a comparison of two extreme groups on the outer ends of the social preference spectrum. Although a hypersensitivity to exclusion in adolescents with a history of rejection is highly consistent with both theoretical accounts of peer relations (Coie 1990; Ladd and Troop-Gordon 2003; Zakriski et al. 1997) and the development of rejection sensitivity (Downey et al. 1998; London et al. 2007), we cannot rule out the possibility that differences between the two groups are partly explained by a hyposensitivity to exclusion in the stably accepted adolescents. That is, a greater exposure to positive peer relations in the stably accepted group could have also had a dampening effect on neural responses to exclusion (Masten et al. 2012). Future research can inform this question by contrasting adolescents with a history of chronic rejection and acceptance with a sample of adolescents with a stable average peer status.

Third, our sample of chronically rejected adolescents contained adolescents with and without a clinical diagnosis of ADHD. Although removing the participants with ADHD from our analyses did not influence our findings, it is important to investigate whether neural responses to exclusion differ between chronically rejected children with ADHD and those without such a diagnosis.

### Conclusions, Implications and Future Directions

To conclude, the present study forms an important first step toward understanding how social exclusion might be experienced differently as a function of an adolescent’s prior peer status history. Using neuroimaging methods we showed that, despite chronically rejected and stably accepted  adolescents reporting similar negative feelings following exclusion, chronically rejected adolescents showed enhanced neural responses to social exclusion and incidental exclusion. Our findings shed light on the processes, occurring at the level of an individual child, through which peer rejection may lead to adverse effects on mental health over time. Crucially, adolescents who have been exposed to chronic peer rejection process the same exclusion experience differently on a neural level compared to adolescents who were not exposed to chronic rejection, which might not be easily captured by self-reports. Longitudinal studies have shown that peer rejection is a very persistent phenomenon, which can generalize across different social contexts. For example, when children with a rejected status in their classroom enter new social situations where they are unknown, they rapidly reestablish a rejected status (Coie and Kupersmidt 1983; Hardy et al. 2002). Consistent with transactional models of peer rejection, children with a heightened neural reactivity to social exclusion might show more pronounced emotional or behavioral reactions to acute rejection experiences (e.g., social exclusion), which could in turn elicit repeated instances of rejection in a new social situation. Thus, sensitivity at the neural level might lead to more negative peer experiences that put adolescents with a history of peer group rejection at greater risk for developing mental health problems. However, more work is needed to definitively pinpoint the psychological processes that heightened neural responses in ACC and aPFC represent and how they affect subsequent psychosocial adjustment.

The current study lays the foundations for future work that can examine how neural responses to social exclusion among rejected adolescents might predict behavioral reactions to exclusion. For example, a heightened responsiveness to exclusion might be related to more aggressive reactions, which could sustain the cycle of repeated instances of rejection and increasingly more behavioral problems in which chronically rejected children might have become trapped. Similarly, a heightened neural reactivity to exclusion might be related to anxious expectations of rejection leading to withdrawal from social interactions. Finally, the current findings can inform interventions aimed at reducing rejected children’s social difficulties by targeting their hypersensitivity to exclusion. Neuroimaging studies of emotional reappraisal have shown that emotion regulation strategies can alter emotion-related neural activity (Ochsner et al. 2012). An interesting future direction would be to test whether emotion regulation strategies could be used to attenuate the heightened neural response to exclusion and how attenuation of the response might influence subsequent acceptance in the peer group. Ultimately, a neurocognitive perspective on the complex interplay between peer relations and psychosocial development may contribute to our understanding of which rejected children are at risk for developing problems and how subjective and neural responses to exclusion might predict adjustment trajectories.

## Electronic supplementary material

Below is the link to the electronic supplementary material.

Supplementary Table 1 (PDF 55 kb)

Supplementary Table 2 (PDF 37 kb)

## References

[CR1] Abrams D, Weick M, Thomas D, Colbe H, Franklin KM (2011). On-line ostracism affects children differently from adolescents and adults. The British Journal of Developmental Psychology.

[CR2] Asher SR, Coie JD (1990). Peer rejection in childhood.

[CR3] Asher SR, Dodge KA (1986). Identifying children who are rejected by their peers. Developmental Psychology.

[CR4] Baumeister RF, Leary MR (1995). The need to belong: desire for interpersonal attachments as a fundamental human motivation. Psychological Bulletin.

[CR5] Boivin M, Hymel S, Bukowski WM (1995). The roles of social withdrawal, peer rejection, and victimization by peers in predicting loneliness and depressed mood in childhood. Development and Psychopathology.

[CR6] Bolling DZ, Pitskel NB, Deen B, Crowley MJ, Mayes LC, Pelphrey KA (2011). Development of neural systems for processing social exclusion from childhood to adolescence. Developmental Science.

[CR7] Botvinick MM, Cohen JD, Carter CS (2004). Conflict monitoring and anterior cingulate cortex: an update. Trends in Cognitive Sciences.

[CR8] Boulton MJ (1999). Concurrent and longitudinal relations between children’s playground behavior and social preference, victimization, and bullying. Child Development.

[CR9] Brett, M., Anton, J.-L., Valabregue, R., & Poline, J.-B. (2002). Region of interest analysis using an SPM toolbox [abstract]. *Presented at the 8th International Conference on Functional Mapping of the Human Brain, June 2-6, 2002, Sendai, Japan. Available on CD-ROM in NeuroImage, 16.*

[CR10] Bukowski WM, Sippola L, Hoza B, Newcomb AF, Cillessen AHN, Bukowski WM (2000). Pages from a sociometric notebook: An analysis of nomination and rating scale measures of acceptance, rejection, and social preference. Recent advances in the measurement of acceptance and rejection in the peer system.

[CR11] Cacioppo, S., Frum, C., Asp, E., Weiss, R. M., Lewis, J. W., & Cacioppo, J. T. (2013). A quantitative meta-analysis of functional imaging studies of social rejection. *Scientific Reports, 3*. 10.1038/srep02027 PMC3761131 24002359

[CR12] Coie JD, Asher SR, Coie JD (1990). Toward a theory of peer rejection. Peer rejection in childhood.

[CR13] Coie JD, Kupersmidt JB, Dodge KA (2004). The impact of negative social experiences on the development of antisocial behavior. Children’s peer relations: From development to intervention.

[CR14] Coie, J. D., & Kupersmidt, J. B. (1983). A behavioral analysis of emerging social status in boys’ groups. *Child Development, 54*, 1400–1416.

[CR15] Crick NR, Dodge KA (1994). A review and reformulation of social information-processing mechanisms in children’s social adjustment. Psychological Bulletin.

[CR16] DeRosier ME, Kupersmidt JB, Patterson CJ (1994). Children’s academic and behavioral adjustment as a function of the chronicity and proximity of peer rejection. Child Development.

[CR17] DeWall CN, Masten CL, Powell C, Combs D, Schurtz DR, Eisenberger NI (2012). Do neural responses to rejection depend on attachment style? An fMRI study. Social Cognitive and Affective Neuroscience.

[CR18] Dodge KA, Lansford JE, Burks VS, Bates JE, Pettit GS, Fontaine R, Price JM (2003). Peer rejection and social information-processing factors in the development of aggressive behavior problems in children. Child Development.

[CR19] Downey G, Lebolt A, Rincon C, Freitas AL (1998). Rejection sensitivity and children’s interpersonal difficulties. Child Development.

[CR20] Eisenberger NI (2012). The pain of social disconnection: examining the shared neural underpinnings of physical and social pain. Nature Reviews Neuroscience.

[CR21] Eisenberger NI, Lieberman MD, Williams KD (2003). Does rejection hurt? An fMRI study of social exclusion. Science.

[CR22] Eisenberger NI, Taylor SE, Gable SL, Hilmert CJ, Lieberman MD (2007). Neural pathways link social support to attenuated neuroendocrine stress responses. NeuroImage.

[CR23] Gerber J, Wheeler L (2009). On being rejected a meta-analysis of experimental research on rejection. Perspectives on Psychological Science.

[CR24] Gunther Moor B, Güroğlu B, Op de Macks ZA, Rombouts SARB, Van der Molen MW, Crone EA (2012). Social exclusion and punishment of excluders: neural correlates and developmental trajectories. NeuroImage.

[CR25] Hardy CL, Bukowski WM, Sippola LK (2002). Stability and change in peer relationships during the transition to middle-level school. The Journal of Early Adolescence.

[CR26] Jiang XL, Cillessen AHN (2005). Stability of continuous measures of sociometric status: a meta-analysis. Developmental Review.

[CR27] Ladd GW (1999). Peer relationships and social competence during early and middle childhood. Annual Review of Psychology.

[CR28] Ladd GW (2006). Peer rejection, aggressive or withdrawn behavior, and psychological maladjustment from ages 5 to 12: an examination of four predictive models. Child Development.

[CR29] Ladd GW, Troop-Gordon W (2003). The role of chronic peer difficulties in the development of children’s psychological adjustment problems. Child Development.

[CR30] Ladd GW, Ettekal I, Kochenderfer-Ladd B, Rudolph KD, Andrews RK (2014). Relations among chronic peer group rejection, maladaptive behavioral dispositions, and early adolescents’ peer perceptions. Child Development.

[CR31] Lelieveld G-J, Gunther Moor B, Crone EA, Karremans JC, van Beest I (2013). A penny for your pain? The financial compensation of social pain after exclusion. Social Psychological and Personality Science.

[CR32] Lieberman MD, Cunningham WA (2009). Type I and type II error concerns in fMRI research: re-balancing the scale. Social Cognitive and Affective Neuroscience.

[CR33] London B, Downey G, Bonica C, Paltin I (2007). Social causes and consequences of rejection sensitivity. Journal of Research on Adolescence.

[CR34] Marks PEL, Babcock B, Cillessen AHN, Crick NR (2013). The effects of participation rate on the internal reliability of peer nomination measures. Social Development.

[CR35] Masten C, Eisenberger N, Borofsky L, Pfeifer J, McNealy K, Mazziotta J, Dapretto M (2009). Neural correlates of social exclusion during adolescence: understanding the distress of peer rejection. Social Cognitive and Affective Neuroscience.

[CR36] Masten C, Eisenberger N, Borofsky L, McNealy K, Pfeifer J, Dapretto M (2011). Subgenual anterior cingulate responses to peer rejection: a marker of adolescents’ risk for depression. Development and Psychopathology.

[CR37] Masten CL, Telzer EH, Fuligni AJ, Lieberman MD, Eisenberger NI (2012). Time spent with friends in adolescence relates to less neural sensitivity to later peer rejection. Social Cognitive and Affective Neuroscience.

[CR38] Ochsner KN, Silvers JA, Buhle JT (2012). Functional imaging studies of emotion regulation: a synthetic review and evolving model of the cognitive control of emotion. Annals of the New York Academy of Sciences.

[CR39] Onoda K, Okamoto Y, Nakashima K, Nittono H, Yoshimura S, Yamawaki S, Ura M (2010). Does low self-esteem enhance social pain? The relationship between trait self-esteem and anterior cingulate cortex activation induced by ostracism. Social Cognitive and Affective Neuroscience.

[CR40] Parker JG, Asher SR (1987). Peer relations and later personal adjustment: are low-accepted children at risk?. Psychological Bulletin.

[CR41] Prinstein MJ, Aikins JW (2004). Cognitive moderators of the longitudinal association between peer rejection and adolescent depressive symptoms. Journal of Abnormal Child Psychology.

[CR42] Rotge, J. Y., Lemogne, C., Hinfray, S., Huguet, P., Grynszpan, O., Tartour, E., . . . Fossati, P. (2014). A meta-analysis of the anterior cingulate contribution to social pain. *Social Cognitive and Affective Neuroscience*. 10.1093/scan/nsu110 PMC4994851 25140048

[CR43] Rubin, K. H., Bukowski, W. M., & Parker, J. G. (2006). Peer interactions, relationships, and groups. In W. Damon, R. M. Lerner (Series Eds.), & N. Eisenberg (Vol. Ed.), *Handbook of child psychology: Social, emotional, and personality development* (pp. 571–645). Hoboken, NJ: John Wiley & Sons.

[CR44] Salmivalli C, Isaacs J (2005). Prospective relations among victimization, rejection, friendlessness, and children’s self- and peer-perceptions. Child Development.

[CR45] Sandstrom MJ (2004). Pitfalls of the peer world: how children cope with common rejection experiences. Journal of Abnormal Child Psychology.

[CR46] Sandstrom MJ, Coie JD (1999). A developmental perspective on peer rejection: mechanisms of stability and change. Child Development.

[CR47] Sandstrom MJ, Cillessen AHN, Eisenhower A (2003). Children’s appraisal of peer rejection experiences: impact on social and emotional adjustment. Social Development.

[CR48] Sanfey AG, Rilling JK, Aronson JA, Nystrom LE, Cohen JD (2003). The neural basis of economic decision-making in the ultimatum game. Science.

[CR49] Sebastian C, Viding E, Williams K, Blakemore SJ (2010). Social brain development and the affective consequences of ostracism in adolescence. Brain and Cognition.

[CR50] Sebastian CL, Tan GCY, Roiser JP, Viding E, Dumontheil I, Blakemore S-J (2011). Developmental influences on the neural bases of responses to social rejection: implications of social neuroscience for education. NeuroImage.

[CR51] Shackman AJ, Salomons TV, Slagter HA, Fox AS, Winter JJ, Davidson RJ (2011). The integration of negative affect, pain and cognitive control in the cingulate cortex. Nature Reviews Neuroscience.

[CR52] Shenhav A, Botvinick MM, Cohen JD (2013). The expected value of control: an integrative theory of anterior cingulate cortex function. Neuron.

[CR53] Somerville LH, Heatherton TF, Kelley WM (2006). Anterior cingulate cortex responds differentially to expectancy violation and social rejection. Nature Neuroscience.

[CR54] Sturaro C, van Lier PAC, Cuijpers P, Koot HM (2011). The role of peer relationships in the development of early school-age externalizing problems. Child Development.

[CR55] Troop-Gordon W, Ladd GW (2005). Trajectories of peer victimization and perceptions of the self and schoolmates: precursors to internalizing and externalizing problems. Child Development.

[CR56] Tzourio-Mazoyer N, Landeau B, Papathanassiou D, Crivello F, Etard O, Delcroix N, Joliot M (2002). Automated anatomical labeling of activations in SPM using a macroscopic anatomical parcellation of the MNI MRI single-subject brain. NeuroImage.

[CR57] van Beest I, Williams KD (2006). When inclusion costs and ostracism pays, ostracism still hurts. Journal of Personality and Social Psychology.

[CR58] van Lier PAC, Koot HM (2010). Developmental cascades of peer relations and symptoms of externalizing and internalizing problems from kindergarten to fourth-grade elementary school. Development and Psychopathology.

[CR59] Vitaro F, Pedersen S, Brendgen M (2007). Children’s disruptiveness, peer rejection, friends’ deviancy, and delinquent behaviors: a process-oriented approach. Development and Psychopathology.

[CR60] Wesselmann ED, Wirth JH, Mroczek DK, Williams KD (2012). Dial a feeling: detecting moderation of affect decline during ostracism. Personality and Individual Differences.

[CR61] Will, G.-J., Crone, E. A., & Güroğlu, B. (2014). Acting on social exclusion: Neural correlates of punishment and forgiveness of excluders. *Social Cognitive and Affective Neuroscience*, nsu045. 10.1093/scan/nsu045 PMC4321620 24652858

[CR62] Williams KD (2007). Ostracism. Annual Review of Psychology.

[CR63] Williams KD, Sommer KL (1997). Social ostracism by coworkers: does rejection lead to loafing or compensation?. Personality and Social Psychology Bulletin.

[CR64] Williams KD, Cheung CKT, Choi W (2000). Cyberostracism: effects of being ignored over the internet. Journal of Personality and Social Psychology.

[CR65] Zadro L, Boland C, Richardson R (2006). How long does it last? The persistence of the effects of ostracism in the socially anxious. Journal of Experimental Social Psychology.

[CR66] Zakriski, A., Jacobs, M., & Coie, J. (1997). Coping with childhood peer rejection. In *Handbook of children’s coping* (pp. 423–451). Springer.

