# Supplementary material for: Chronic Childhood Peer Rejection is Associated with Heightened Neural Responses to Social Exclusion During Adolescence
Source: J Abnorm Child Psychol. 2015 Mar 12;44(1):43–55. doi: 10.1007/s10802-015-9983-0 (PMC4715124; doi:10.1007/s10802-015-9983-0)
Supplement: Supplementary file 1 — (PDF 55 kb) [file 10802_2015_9983_MOESM1_ESM.pdf]

Supplementary Table 1

Assessment of Need Satisfaction and Mood following Inclusion, Exclusion and 30 minutes after exclusion (when participants came out of the scanner). All items were rated on a scale from 1 ('not at all') to 5 ('very much'). (R) = reversed scored.

Construct	Item
**Need satisfaction**	
Belonging	1. I had the feeling that I belonged to the group during the game.
	2. During the game I felt connected with one or more players.
Control	3. I had the feeling that the other players decided everything. (R)
	4. I felt in control over the game.
Self esteem	5. I felt good about myself.
	6. My self-esteem was high.
Meaningful existence	7. During the game it felt as if my presence was not meaningful. (R)
	8. I had the feeling that my presence during the game was important.
**Mood**	During the game I felt:
	1. Good
	2. Bad (R)
	3. Happy
	4. Sad (R)
	5. Relaxed
	6. Tense (R)
	7. Friendly
	8. Unfriendly (R)
