# Supplementary material for: Chronic Childhood Peer Rejection is Associated with Heightened Neural Responses to Social Exclusion During Adolescence
Source: J Abnorm Child Psychol. 2015 Mar 12;44(1):43–55. doi: 10.1007/s10802-015-9983-0 (PMC4715124; doi:10.1007/s10802-015-9983-0)
Supplement: Supplementary file 2 — (PDF 37 kb) [file 10802_2015_9983_MOESM2_ESM.pdf]

Supplementary table 2

Brain regions revealed by whole-brain contrasts during the Cyberball game across the sample (all thresholded p < .001 uncorrected, > 10 voxels).

Anatomical region	L/R	Voxels	z	MNI coordinates		
				x	y	z
**Social exclusion (Exclusion: not receiving the ball > Inclusion: receiving the ball)**						
Calcarine gyrus/Cuneus	L	1376	6.70	-12	-97	4*
			6.63	-12	-88	1*
			5.70	12	-88	1*
Caudate	L	82	5.26	-6	17	-2*
			3.17	9	8	1*
Ventral Anterior Cingulate cortex/	L	174	4.62	-12	47	1*
Medial Prefrontal cortex			4.10	0	50	-5*
			3.99	12	47	-2*
Precentral gyrus	L	64	4.57	-39	-16	40*
Inferior Frontal gyrus	R	47	4.37	27	32	-11*
			3.76	36	35	-11*
Inferior Frontal gyrus (vlFPC)	L	89	4.28	-45	32	-8*
			4.12	-24	32	-11*
			3.84	-36	32	-11*
Superior Temporal gyrus/	R	46	3.76	66	-10	4*
Posterior Insula			3.49	42	-19	4*
			3.37	54	-10	7*
Superior Frontal gyrus (dmPFC)	L	22	3.63	-6	47	46*
Superior Temporal gyrus	L	13	3.54	-66	-28	4*
**Social exclusion (Exclusion: not receiving the ball > Inclusion: not receiving the ball)**						
Caudate	L/R	79	4.00	-6	17	-2
			3.86	12	8	1
			3.84	6	17	-2
Posterior Cingulate	L/R	56	3.88	-6	-34	25
			3.88	0	-34	19

Ventral Anterior Cingulate cortex/	L/R	38	3.72	-6	44	1
Medial Prefrontal cortex			3.19	12	47	-2
**Incidental exclusion (Inclusion: not receiving the ball > Inclusion: receiving the ball)**						
Cuneus/Calcarine gyrus	L	648	6.66	-12	91	1*
			6.12	12	-85	4*
			3.91	27	-79	5*
Paracentral Lobule/Precentral gyrus	L	350	4.90	-6	-34	61*
			4.63	15	-31	64*
Poscentral gyrus			4.49	-18	-34	76*
Posterior Insula/		88	4.69	36	-13	19*
Precentral gyrus			4.00	39	-16	37*
Precentral gyrus	L	24	4.58	-39	-16	40*
Fusiform gyrus	R	106	4.47	27	-40	-14*
			4.09	33	-40	-14*
Inferior Frontal gyrus	L	19	4.18	-39	32	-11*
Middle Temporal gyrus	L	20	3.88	-60	-7	-17*
Postcentral gyrus	R	10	3.87	63	-7	22*
Hippocampus	L	16	3.87	-30	-10	-23*
Inferior Frontal gyrus	R	18	3.75	36	32	-11*
Medial Prefrontal cortex	L	12	3.75	-9	50	-5*
Inferior Frontal gyrus (vlPFC)	L	13	3.66	-54	29	7*
Parahippocampal gyrus/	L	26	3.55	-24	-37	-14*
Fusiform gyrus			3.28	-33	-31	-17*

Note. L/R=Left/Right; k=cluster size in 3×3×3mm voxels; Z=z-score; MNI coordinates =xyz voxel coordinates in MNI space of the peak voxel. Ball = events on which participants received the ball; No ball = events where participants did not receive the ball. * = also significant using FDR correction, $p < .05$, > 10 voxels). dmPFC = Dorsomedial prefrontal cortex; vlPFC = Ventrolateral prefrontal cortex
